# Basaloid Squamous Cell Carcinoma of the Ethmoid Sinus with Invasion into the Skull Base Treated with Craniofacial Resection and Adjuvant Intensity-Modulated Radiation Therapy: A Case Report

**DOI:** 10.7759/cureus.421

**Published:** 2015-12-21

**Authors:** Karine A Al Feghali, Henri Traboulsi, Bassem Youssef

**Affiliations:** 1 Radiation Oncology Department, American University of Beirut Medical Center; 2 Pediatric Otolaryngology, Detroit Medical Center

**Keywords:** basaloid squamous cell carcinoma, ethmoid sinus, sinonasal tumors, intensity modulated radiation therapy

## Abstract

Basaloid squamous cell carcinoma (BSCC) is a rare variant of squamous cell carcinoma (SCC), which is highly aggressive, with a tendency for multifocality, local invasion, and with a high metastatic potential. Less than forty cases of BSCC of the sinonasal tract have been reported in the literature, and no reports were found on sinonasal BSCC arising from the ethmoid sinus. We report the case of a 78-year-old man who presented with BSCC arising from the ethmoid sinus with extensive bone destruction and intracranial extension. He was treated with craniofacial resection followed by adjuvant intensity-modulated radiation therapy to the tumor bed (60 Gy in 30 fractions), and the upper neck lymph nodes (50 Gy in 25 fractions). At the patient’s last follow-up, four months after diagnosis, there was no evidence of disease. Aggressive management using craniofacial resection followed by adjuvant radiation therapy with or without radiosensitizing chemotherapy seems to be a reasonable approach to this challenging disease.

## Introduction

The first reports of basaloid squamous cell carcinoma (BSCC) date back to 1986 [1]. It is a rare variant of SCC, which is highly aggressive, with a tendency for multifocality, local invasion, and with a high metastatic potential. It has been shown to have a predilection for the upper aerodigestive tract: mainly the larynx, hypopharynx, and oropharynx [2]. It is, however, not limited to the head and neck region and has been reported in the gastrointestinal and genitourinary systems as well [2]. Less than forty cases of BSCC of the sinonasal tract have been reported in the literature [3-10], and reports of sinonasal BSCC arising from or involving the ethmoid sinus are even more scarce [5, 7]. Data on the management of this rare entity is limited. 

## Case presentation

A 78-year-old man presented with an eight-month history of nasal obstruction and self-resolving episodes of epistaxis. The patient denied any history of tobacco or alcohol use and had no prior history of radiation. He was previously treated with partial colectomy and adjuvant chemotherapy for colon adenocarcinoma.

On physical examination, the patient had a Karnofsky performance status (KPS) of 90%, ECOG (Eastern Cooperative Oncology Group) of I, an erythematous nasal mass filling both nasal cavities, and no palpable cervical lymphadenopathy. There was no evidence of neurological deficits, and cranial nerves, 2 to 12, were intact.

**Figure 1:**
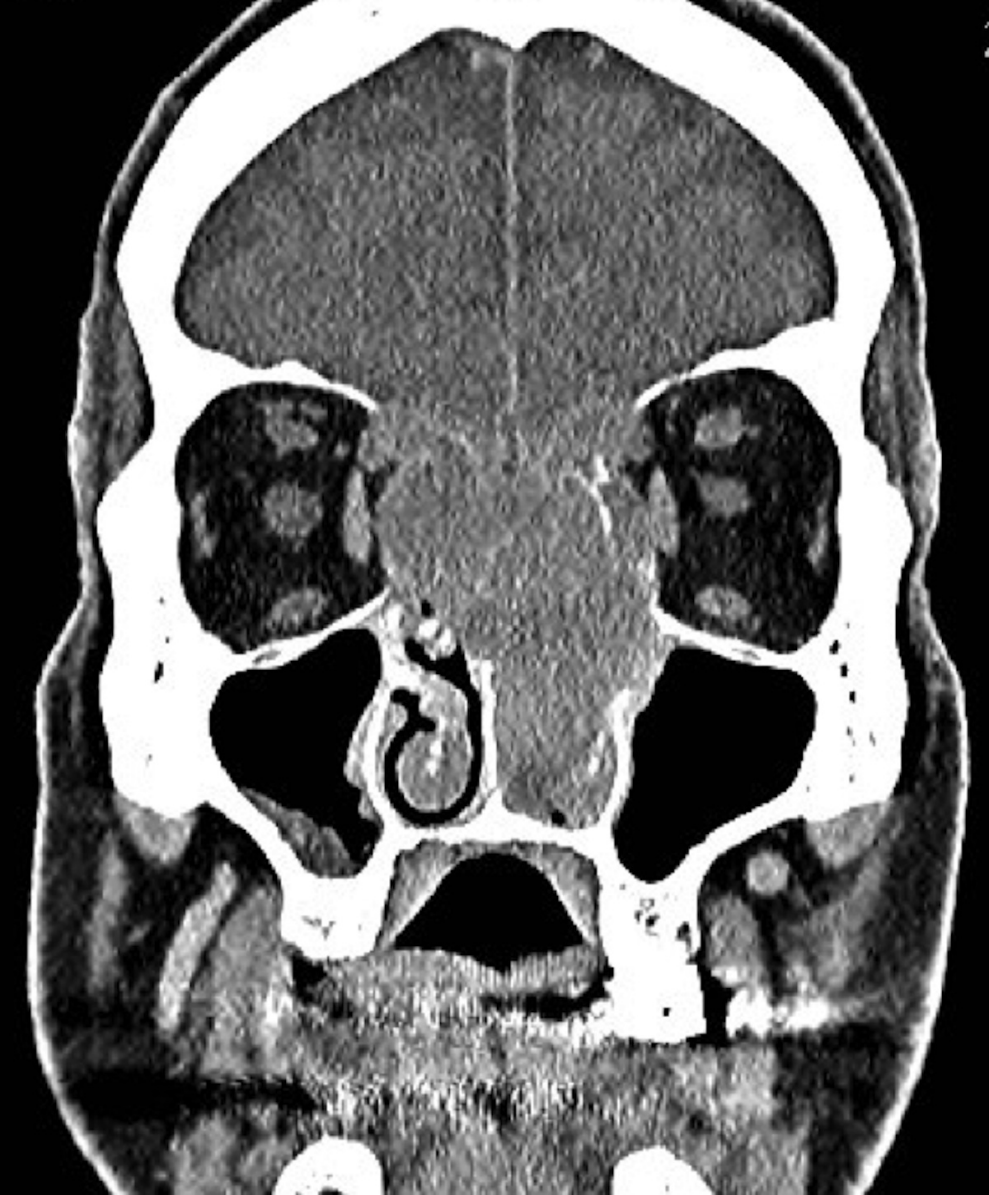
Contrast-enhanced computed tomography scan Coronal cut showing the lesion eroding the bilateral lamina papyracea and the cribriform plate and encroaching on the medial recti muscles.

**Figure 2:**
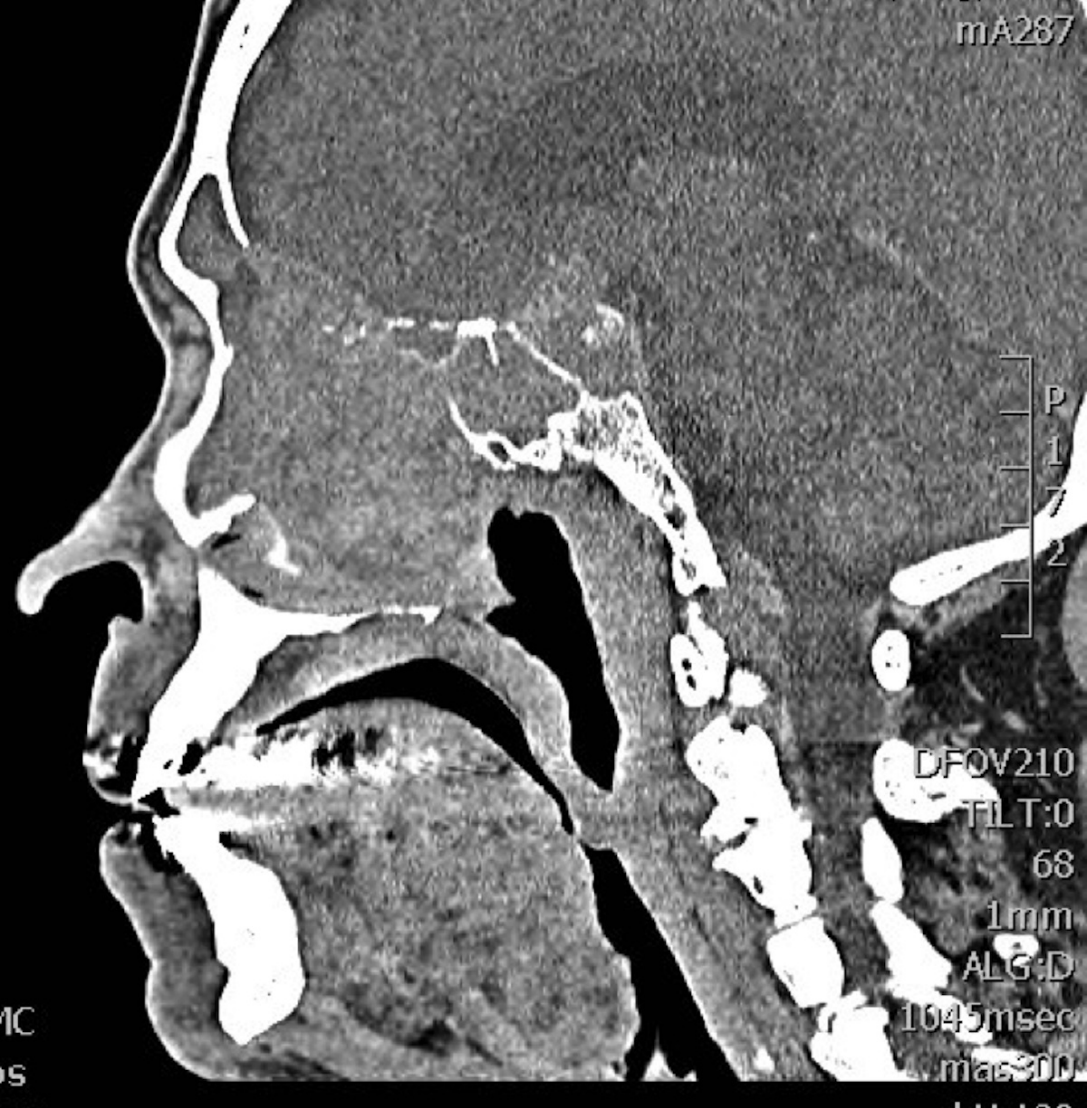
Contrast-enhanced computed tomography scan Sagittal cut showing the lesion involving the ethmoid sinuses, invading the nasal cavity and septum, the frontal and sphenoid sinuses, with base of skull erosion and intracranial extension.

**Figure 3:**
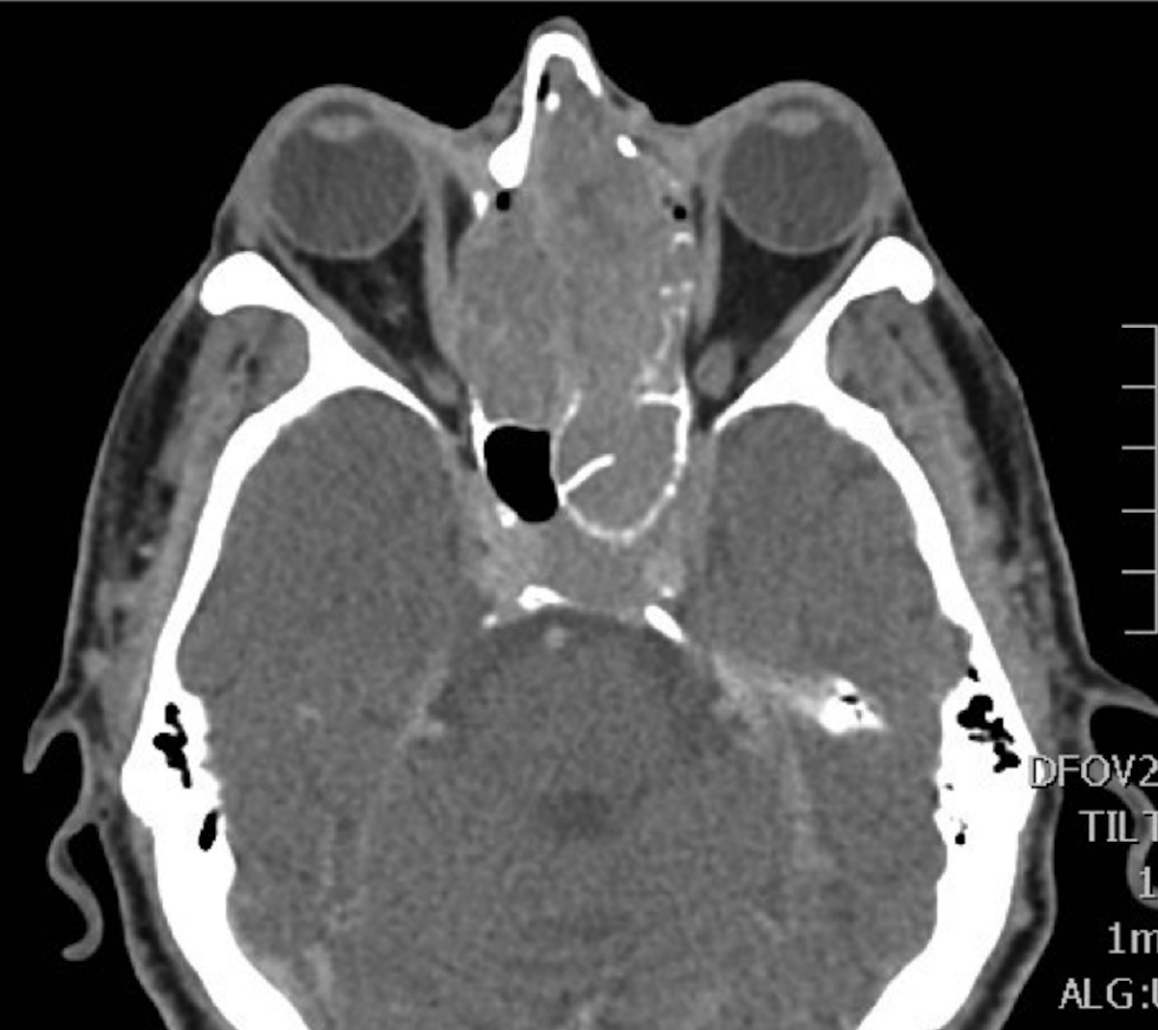
Contrast-enhanced computed tomography scan Axial cut showing the lesion extending into the left sphenoid sinus, involving the cavernous sinus and engulfing the left internal carotid artery.

A computed tomography (CT) scan of the head and sinuses revealed a large (5 x 4 x 6.5 cm) enhancing soft tissue lesion filling the left nasal cavity, expanding to destroy the ethmoid air cells bilaterally and obliterating the frontal sinuses. The mass was seen eroding both lamina papyracea and causing mass effect on the medial rectus muscles bilaterally. It was extending superiorly and eroding the planum sphenoidale. Posteriorly, the mass was invading the left sphenoid sinus and extruding into the pituitary fossa, and ultimately extending superiorly to the level of the third ventricle and causing dilatation of the lateral ventricles. The lesion was involving the cavernous sinus and engulfing the cavernous portion of the left internal carotid artery as well as the first segment of the left middle cerebral artery (Figures 1-3). Magnetic resonance imaging of the head and neck revealed extension to the anterior cranial fossa through the anterior aspect of the cribriform plate.

Informed patient consent was obtained. No identify patient information is disclosed in this report.

Transnasal biopsy of the lesion revealed high-grade carcinoma with clear cell, basaloid, and squamous features, most consistent with poorly differentiated basaloid squamous cell carcinoma. Tumor cells were positive for CK7, CK 5/6, and p63. They were negative for S-100 and synaptophysin.

CT scan of the chest and a fluorodeoxyglucose (FDG)-positron emission tomography (PET) scan revealed no evidence of regional or distant metastasis. Clinical staging of this tumor, based on the American Joint Committee on Cancer (AJCC)  TNM staging system for the nasal cavity and paranasal sinuses (7th edition, 2010), was T_4_N_0_M_0_.  

The tumor was removed by combined endoscopic and open craniofacial approaches. Final histopathological examination of the lesion showed a poorly differentiated basaloid squamous cell carcinoma with positive resection margins. The patient had a smooth postoperative course with no significant complications.

**Figure 4:**
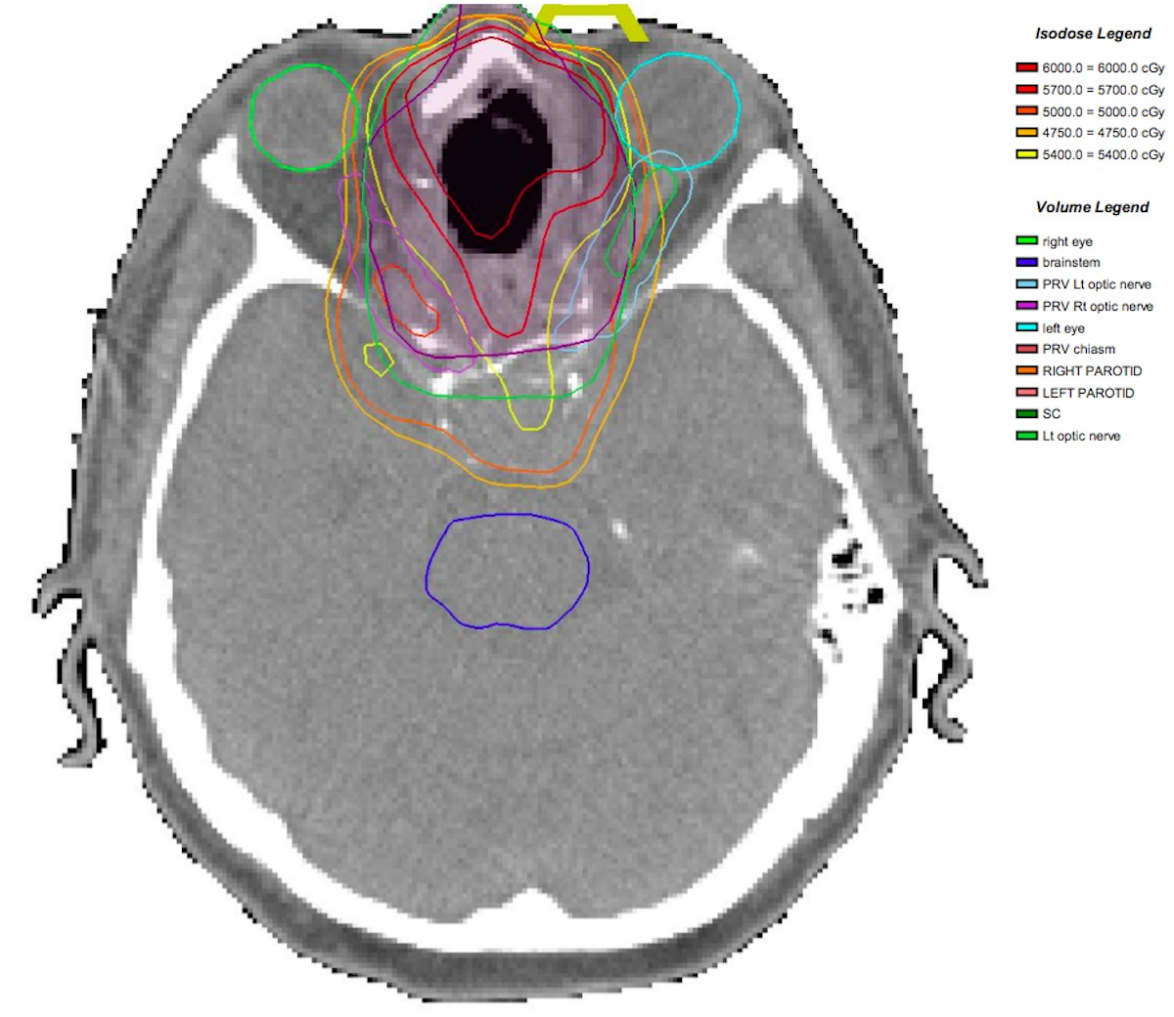
Intensity-modulated radiation therapy plan Axial cut showing the isodose lines curving around the optic nerves. The tumor bed was treated to 60 Gy in 30 fractions.

Adjuvant intensity-modulated radiation therapy (IMRT) was administered 31 days after surgery. The tumor bed received a total dose of 60 Gy in 30 fractions, and the upper neck lymph nodes, mainly the retropharyngeal, level IB, and II lymph nodes received a total dose of 50 Gy in 25 fractions (Figure 4). During radiation treatment, the patient developed a Grade 2 dermatitis and oral mucositis, conjunctivitis, odynophagia, and fatigue. No Grade 3 or higher toxicities were reported.

At the patient’s last follow-up, four months after diagnosis, there was no evidence of disease.

## Discussion

Around 40 sinonasal BSCC cases are reported in the literature. Patients most commonly present with a facial mass, nasal obstruction, epistaxis [3, 5-7, 9], and less frequently, with visual symptoms (diplopia and blurred vision) [3]. Sinonasal BSCC cases reported in the literature often evoke bone erosion [4, 7], dural invasion [7], and intracranial extension (two of 14 cases in a review by Wieneke, et al. [3]) testifying to the locally aggressive nature of this rare entity. Sinonasal BSCC usually presents with an advanced clinical stage at initial diagnosis [2]. Many patients have systemic metastases at diagnosis, mostly to the lung, liver, or bone (five of 14 cases in Wieneke, et al.’s review [3]), and the spread can sometimes be rapidly fatal [10].

The optimal management of sinonasal BSCC remains to be elucidated, as there exists no high-level evidence to this regard. It is usually thought to require multimodality treatment. The first treatment modality has usually consisted of surgical resection [3, 7, 9]. Adjuvant treatment, consisting of radiation therapy with or without chemotherapy, has been advocated due to the aggressiveness of this disease [3, 7]. Intensity-modulated radiation therapy (IMRT), used in our case, offers good conformality, the advantage of sparing critical structures (such as optic nerves and chiasm), as well as the opportunity to escalate the dose in high-risk areas, as in the setting of close or positive margins. In some instances, neoadjuvant chemoradiotherapy [4] or radiation therapy [8] at doses of 40-45 Gy have been administered to render the tumor resectable [4]. We found a few reported cases treated with definitive chemoradiotherapy [5-7], one of which used proton beam therapy to 70 cobalt Gy equivalent (CGE), with concomitant single-agent, high-dose cisplatin (100 mg/m2) on days 1, 22 and 43 [5]. The patient reported in that paper was free of disease at his 24-month follow-up. Although appealing, the limited availability of proton beam therapy makes this approach limited to a few centers around the world. Adding concomitant chemotherapy to adjuvant radiation therapy, i.e. high-dose cisplatinum, as a radiosensitizer and to mitigate the occurrence of distant metastasis, might be beneficial, in light of this tumor’s significant potential for local and distant recurrence.

## Conclusions

We report a case of locally invasive sinonasal BSCC treated with craniofacial resection, followed by adjuvant intensity-modulated radiation therapy. This case is one of the very few reported cases of ethmoid sinus BSCC in the literature, and the second reported case of sinonasal BSCC that details radiation therapy dose and volumes. Due to the rarity of sinonasal BSCC, the standard of care has not been established, but aggressive management using craniofacial resection followed by adjuvant intensity-modulated radiation therapy with or without chemotherapy seems to be an acceptable option. 
